# Coupling of Redox and Structural States in Cytochrome P450 Reductase Studied by Molecular Dynamics Simulation

**DOI:** 10.1038/s41598-019-45690-2

**Published:** 2019-06-27

**Authors:** Mikuru Iijima, Jun Ohnuki, Takato Sato, Masakazu Sugishima, Mitsunori Takano

**Affiliations:** 1 Department of Pure and Applied Physics, Waseda University, Tokyo, 169-8555 Japan; 2 Department of Medical Biochemistry, Kurume University School of Medicine, Kurume, Fukuoka 830-0011 Japan

**Keywords:** Bioenergetics, Biophysical chemistry

## Abstract

Cytochrome P450 reductase (CPR) is the key protein that regulates the electron transfer from NADPH to various heme-containing monooxygenases. CPR has two flavin-containing domains: one with flavin adenine dinucleotide (FAD), called FAD domain, and the other with flavin mononucleotide (FMN), called FMN domain. It is considered that the electron transfer occurs via FAD and FMN (NADPH → FAD → FMN → monooxygenase) and is regulated by an interdomain open-close motion. It is generally thought that the structural state is coupled with the redox state, which, however, has not yet been firmly established. In this report, we studied the coupling of the redox and the structural states by full-scale molecular dynamics (MD) simulation of CPR (total 86.4 μs). Our MD result showed that while CPR predominantly adopts the closed state both in the oxidized and reduced states, it exhibits a tendency to open in the reduced state. We also found a correlation between the FAD-FMN distance and the predicted FMN-monooxygenase distance, which is embedded in the equilibrium thermal fluctuation of CPR. Based on these results, a physical mechanism for the electron transfer by CPR is discussed.

## Introduction

The high-energy electron in the reduced-form nicotinamide adenine dinucleotide phosphate (NADPH) provides the driving force for a large number of biochemical reactions in living organisms^1^. Cytochrome P450 reductase (CPR) is the key protein that receives the high-energy electron from NADPH and distributes it selectively to heme-containing monooxygenases such as cytochrome P450^2^ and heme oxygenase (HO)^3^; upon receiving the electrons from CPR, cytochrome P450 and HO become capable of metabolizing drugs^2^ and decomposing toxic free hemes^4^, respectively. Furthermore, CPR can be used in the anticancer therapy where anticancer prodrugs are locally activated by the reducing power of CPR^5^.

CPR has two flavin-containing domains, one with flavin adenine dinucleotide (FAD), called “FAD domain”, and the other with flavin mononucleotide (FMN), called “FMN domain”, and these two domains are connected by a “connecting domain^6^”. The FAD domain contains the NADPH binding site close to the embedded FAD cofactor, which is suited for the electron transfer (hydride transfer) from NADPH to FAD^6^. The crystal structure by Wang *et al*.^7^ showed that CPR adopts a “closed form” where FAD and FMN cofactors are situated in close proximity to each other so that the electron transfer from FAD to FMN would become efficient. On the other hand, Hamdane *et al*. found structural polymorphism in the crystal structure of a mutant of CPR, where the FMN domain is positioned away from the FAD domain^8^, which is referred to as the “open form”. The open form appeared to be suited for the intermolecular interaction with monooxygenase, facilitating the electron transfer from FMN to the heme in the monooxygenase^8^. Indeed, this mutant CPR was found to strongly interact with HO, and the crystal structure of the mutant CPR in complex with HO was solved by Sugishima *et al*.^9^ where CPR adopts an open form similar to that observed in the uncomplexed CPR. Recently, Freeman *et al*. reported the solution structure of another CPR mutant in complex with cytochrome *c* using small-angle neutron scattering (SANS)^10^, which indicated the same binding mode as observed in the crystal structure of the CPR-HO complex.

Therefore, CPR is considered to regulate the electron transfer from FMN to monooxygenases via the structural state change, most likely the open-closed-like interdomain rearrangement, that alters the binding affinity with the monooxygenases^11^. Importantly, the structural state of CPR is considered to be coupled with its redox state. Indeed, several experimental observations indicated that CPR adopts the closed state in the oxidized state whereas it adopts the open state in the reduced state^12–15^. However, some experimental observations^16,17^ suggested that the redox-state-dependent structural change is only marginal compared to the structural change that is anticipated from the closed and the open forms as observed in the crystal structures. The coupling of the redox and the structural states in CPR, thus, has not yet been firmly established. To address this issue, we conducted molecular dynamics (MD) simulation and studied how the structural state of CPR is affected by its redox state. So far, only a few MD simulations have been done to study the structural state of CPR^18,19^. To the best of our knowledge, the present study is the first report on full-scale MD simulation (total 86.4 μs) of CPR toward elucidating the coupling of the redox and the structural states.

## Results

**Figure 1:**
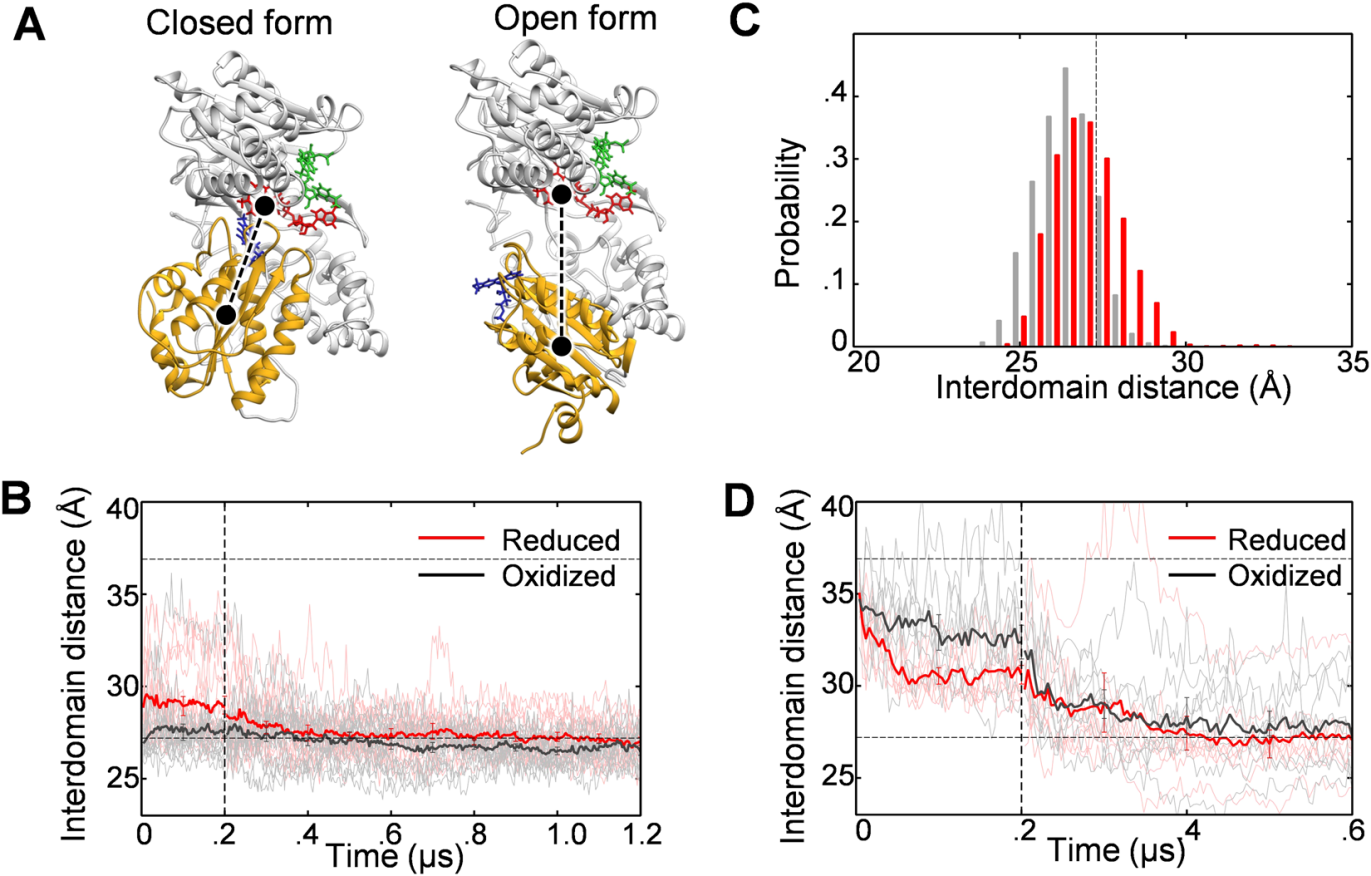
Redox-state dependence of FAD-FMN interdomain distance. (**A**) Crystal structures in the closed form^7^ and the open form^8^; FAD domain (including the connecting domain): gray, FMN domain: yellow, FAD (cofactor): red, FMN (cofactor): blue, and NADP^+^: green. Black circle indicates the center of mass of each domain. (**B**) Average time course of the interdomain distance, starting from the closed form [0–0.2 μs: cMD, 0.2–1.2 μs: aMD; 16 individual time courses are also shown (oxidized: gray, reduced: pink)]. Horizontal lines indicate the values for the crystal structures. (**C**) Probability distributions of the interdomain distance for the oxidized (gray) and reduced (red) states calculated using reweighted data for the last 0.5 μs (see Methods for the reweighting). (**D**) Average time course of the interdomain distance, starting from the open form [0–0.2 μs: cMD, 0.2–0.6 μs: aMD; 8 individual time courses are also shown (oxidized: gray, reduced: pink)].

To investigate the coupling of the redox and the structural states, we first studied the domain-level open-closed-like structural change as inferred from the crystal structures^7,8^ (Fig. 1A). In Fig. 1B, the time courses for the distance between FAD and FMN domains are displayed; in this case, MD simulations were started from the closed form^7^, and 16 independent 0.2-μs conventional MD (cMD) runs were conducted for each redox state (oxidized or reduced). cMD runs were followed by 1-μs accelerated MD (aMD)^20^ runs. In the cMD period, the average time course of the interdomain distance for the reduced state (red) showed an immediately increase. However, it did not show further increase that is expected if CPR adopts the open form. To see whether or not CPR becomes more opened on a longer time scale, we employed the aMD technique^20^ after 0.2 μs. The time course data for the reduced state in the aMD period (0.2–1.2 μs) demonstrates that the interdomain distance did not increase but gradually decreased to the value of the closed form. The interdomain distance for the oxidized state also remained near the value of the closed form. These results indicate that the closed state of CPR is intrinsically stable. However, the probability distributions of the interdomain distance that were calculated using the data for the last 0.5 μs (Fig. 1C) show that the distribution for the reduced state is shifted toward the open form compared to that for the oxidized state, indicating that there is a coupling of the redox and the structural states. To see the influence of the initial structure, we started the MD simulations from the open form^8^. As seen in Fig. 1D, the interdomain distance for both the oxidized and the reduced states largely decreased in the cMD period (first 0.2 μs), and continued to decrease in the subsequent aMD period (0.2–0.6 μs) to the value of the closed form, again indicating the intrinsic stability of the closed state. However, the closed state observed in this case (Fig. 1D) was not the same as that observed in the simulations starting from the closed form (Fig. 1B,C); the interdomain distance exhibited larger fluctuation in this case, so the coupling of the redox and the structural states was obscured.

**Figure 2:**
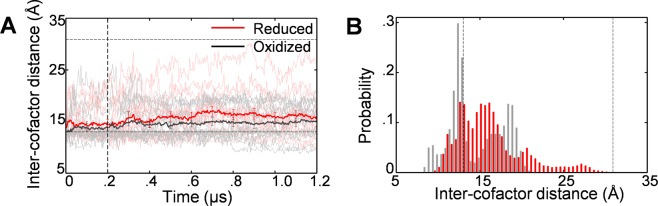
Redox-state dependence of FAD-FMN inter-cofactor distance (the distance between the center of mass of the alloxazine ring of FAD cofactor and that of the FMN cofactor). (**A**) Average time course of the inter-cofactor distance, starting from the closed form [0–0.2 μs: cMD, 0.2–1.2 μs: aMD; 16 individual time courses are also shown (oxidized: gray, reduced: pink)]. Horizontal lines indicate the values for the crystal structures. (**B**) Probability distributions of the inter-cofactor distance for the oxidized (gray) and reduced (red) states calculated using reweighted data for the last 0.5 μs.

We further analyzed the redox-state dependence of the inter-cofactor distance between FAD and FMN. Since it is the alloxazine rings in the cofactors that are directly involved in the electron transfer^6^, we monitored the distance between the center of mass of the alloxazine ring of FAD and that of FMN (Fig. 2). In the cMD period (first 0.2 μs), the average inter-cofactor distance slightly increased in the reduced state, as was observed in the interdomain distance. In the subsequent aMD period, the average inter-cofactor distance in the reduced state further increased, which was not observed in the interdomain distance, and one trajectory came close to the value of the open form. Although the distribution of the inter-cofactor distance (Fig. 2B) was more widespread than that of the interdomain distance because of the thermal fluctuations of the bound cofactors, the redox-state dependence can be seen in the inter-cofactor distance as well as in the interdomain distance.

**Figure 3:**
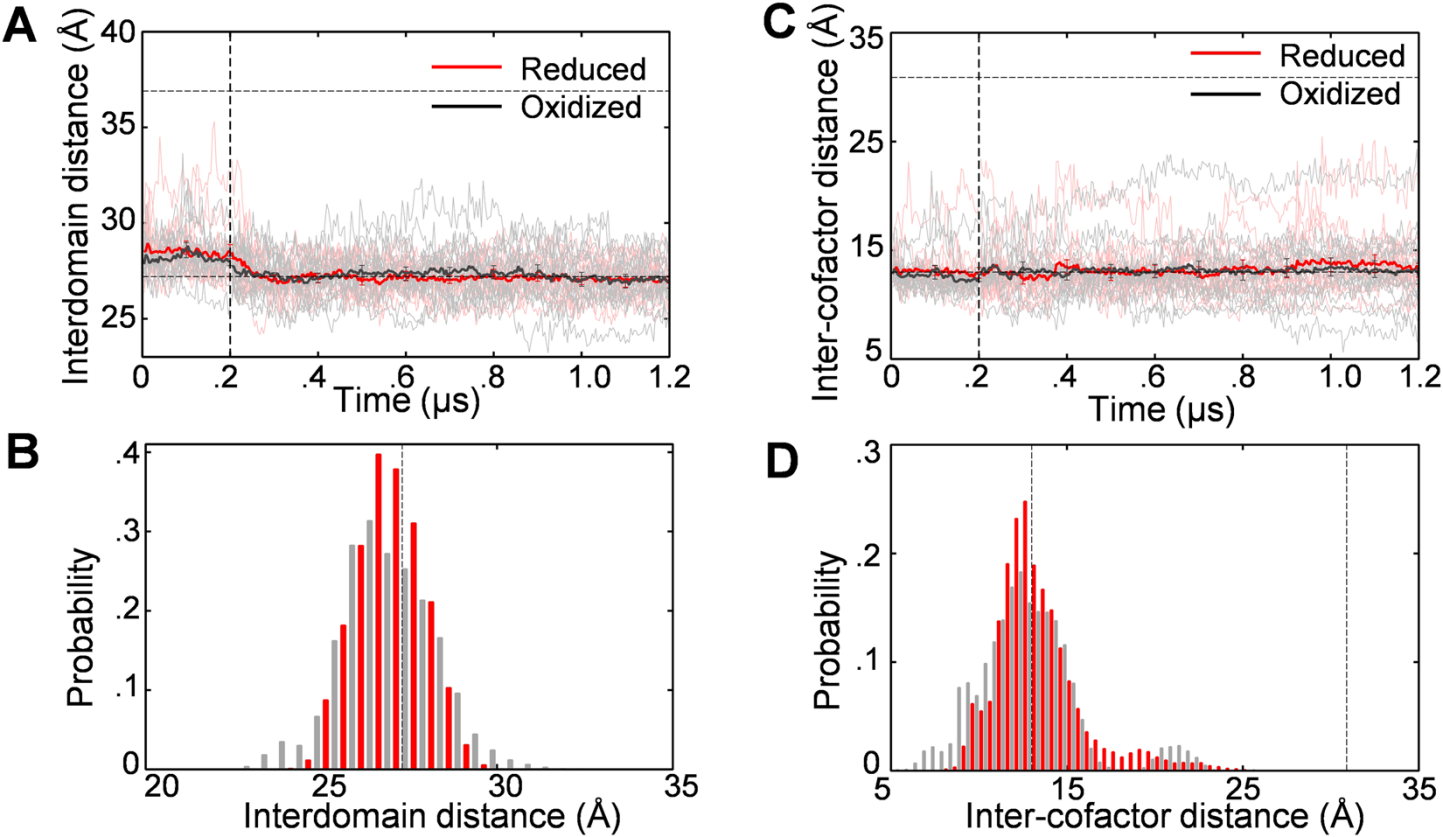
Effect of the bound NADP^+^ on the redox-state dependence of the structural state. The same as in Figs 1B,C and 2A,B except that the bound NADP^+^ was absent.

In addition to the redox state, the bound NADP^+^ is considered to have an influence on the structural state of CPR^12–15,21,22^. We then examined the structural relaxation of CPR in the absence of the bound NADP^+^. In Fig. 3, the time courses and the probability distributions for the interdomain and inter-cofactor distances are shown for the oxidized and the reduced states. In the absence of the bound NADP^+^, the closed form was intrinsically stable in both the oxidized and the reduced states, as was the case in the presence of the bound NADP^+^. However, the redox-state dependence that was observed in the presence of the bound NADP^+^ disappeared in the absence of the bound NADP^+^, suggesting that the bound NADP^+^ is involved in the coupling of the redox and the structural states. We hereafter focus our attention on the results in the presence of the bound NADP^+^.

**Figure 4:**
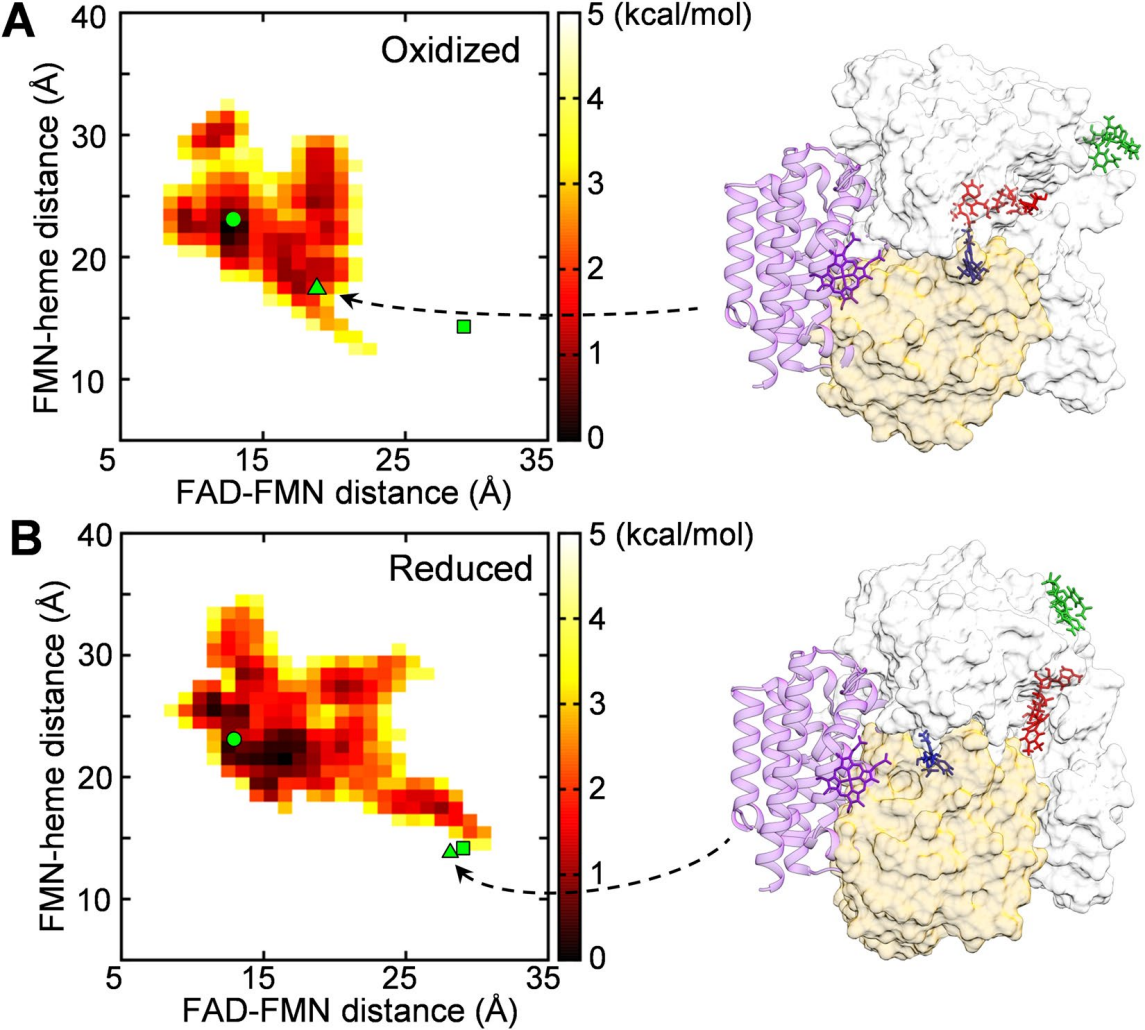
Free-energy landscape depicted in the 2D space constructed by the FAD-FMN distance and the FMN-heme distance: (**A**) free-energy landscape for the oxidized state, and (**B**) that for the reduced state. The free energies were calculated by −*k*_B_*T* ln *P*, where *P* represents the probability density obtained from the reweighted data for the last 0.5-μs aMD, and were adjusted so that the lowest free-energy value becomes zero. FMN-heme distance is a predicted one using the CPR-HO complex structure^9^ (see text), and the center-of-mass distance between the alloxazine ring of FMN and the heme in HO was used. The circle and the square represent the crystal structure of the closed^7^ and that of the open form^9^, respectively. MD snapshot structures (triangles in **A** and **B**) are shown in the right (the heme and HO are shown in purple; other coloring and the view angle are the same as in Fig. 1A).

There is an expectation that the observed structural state of CPR in the reduced state, where the inter-cofactor distance is increased, may exhibit a suitability for the electron transfer from the FMN cofactor to the heme in monooxygenase. We then investigated the distance between the FMN cofactor and the heme using the complex structure of CPR and heme oxygenase (HO)^9^. The CPR-HO complex structure indicates that the FMN cofactor that is buried in the closed form^6,7^ is exposed to the interaction interface with HO, and both the FAD and FMN domains contribute to the interaction with HO. In Fig. 4, we show the correlation between the FAD-FMN distance and the FMN-heme distance by depicting 2D free-energy landscape. Since HO was not included in our MD simulation, we predicted the distance between the FMN cofactor and the heme using the CPR-HO complex structure; the position of HO and heme relative to CPR was determined by superimposing the FAD domain of a MD snapshot structure onto the FAD domain of the CPR-HO complex structure^9^. The low free-energy region in Fig. 4 indicates that the FMN-heme distance has a tendency to decrease as the FAD-FMN distance increases (the correlation coefficient between the FAD-FMN and the FMN-heme distances is −0.21 for the oxidized state and −0.41 for the reduced state). Furthermore, the low free-energy region in the reduced state extended to the region near the crystal structure of the complex (the square and the triangle in the landscape correspond to the crystal structure of the complex and a MD snapshot structure, respectively).

**Figure 5:**
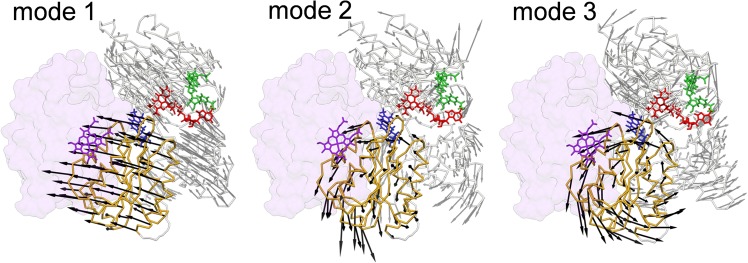
Principal modes of CPR obtained by the principal component analysis for the thermal fluctuation in the reduced state. Three largest-amplitude principal modes (mode 1, 2 and 3) are shown. Each principal mode is represented by a set of vectors (arrows). To highlight the movement of the FMN domain (black arrows), the principal modes are depicted after superimposing the FAD domain onto the CPR-HO complex structure.

We then performed the principal component analysis (PCA) for the thermal fluctuation in the reduced state. In PCA, the eigenvectors of the 3*N* × 3*N* variance-covariance matrix for the equilibrium thermal fluctuation (*N* is the number of the C_*α*_ atoms in CPR), referred to as the “principal modes” representing the collective motions of the atoms in CPR, are obtained. The principal modes thus obtained are shown in Fig. 5 for those with largest amplitudes. Those principal modes indicate that CPR possesses intrinsic thermal fluctuation by which the FMN cofactor comes close to the heme of HO, facilitating the electron transfer from the FMN cofactor to the heme of HO.

## Discussion

Our MD simulation indicated that the closed state is intrinsically stable. Although the open form as found in the crystal structure^8^ was not observed in our MD simulation, the interdomain distance and the inter-cofactor distance between FAD and FMN showed a tendency to increase in the reduced state, suggesting that the structural state is coupled with the redox state. The redox-state dependent structural state has been investigated experimentally^12–15,17,23–25^. It was shown that CPR predominantly adopts the closed state in the oxidized state^12,14,15,25^ and becomes opened in the reduced state^12,14,15^. The observation in our MD simulation is in accord with these experimental observations, even though the observed tendency to open in the reduced state was rather weak in our MD simulation. The weak tendency to open in the reduced state, on the other hand, seems to be well in accordance with the recent FRET experiment by Kovrigina *et al*.^17^. In the present MD study, we considered the two end states of CPR: the fully oxidized state (FAD and FMN are both oxidized), and the fully (4-electron) reduced state (FAD and FMN are both 2-electron reduced). While the 1- to 3-electron reduced states are considered as the major redox states of CPR *in vivo*, the 4-electron reduced state can also be realized at high NADPH concentrations^6^, which is the case in the experiment by Kovrigina *et al*.^17^. Therefore, the reduced state employed in our MD study is considered to be the same as in the FRET study^17^. In addition, the recent SANS experiment by Freeman *et al*.^15^ indicated that the 4-electron reduced state is more opened (extended) than the 1- to 3-electron reduced states, whereas the 1- to 3-electron reduced states are more opened than the fully oxidized (0-electron) state. Therefore, our MD study is considered to capture the two end states of CPR in terms of the structural state as well. Although there still remains a concern about the statistical uncertainty, our MD simulation, with total 86.4 μs MD data in combination with the aMD technique, is expected to have captured the coupling of the redox and the structural states. To obtain the whole spectrum of the redox-structural state coupling, MD simulations for the intermediate redox states (1- to 3-electron reduced states) should be conducted, keeping in mind that a recent single-protein tracking experiment^26^ indicated that CPR in the 2-electron reduced state binds to a redox partner (cytochrome P450) more strongly than CPR in the fully oxidized state and furthermore the binding constant is at the same level as in the fully reduced state.

With regard to the effect of the bound NADP^+^ on the structural state of CPR, the currently prevailing view is that CPR adopts the closed form in the NADP^+^ bound state^12–15,22^, which was not observed in our MD simulation. On the other hand, our MD result that the interdomain distance slightly increased in the presence of the bound NADP^+^ is consistent with the surface plasmon resonance study^21^ where NADP^+^ binding to CPR was shown to strengthen the binding affinity of CPR with HO, suggesting that CPR becomes opened upon NADP^+^ binding.

Based on our MD results, we can envisage a physical mechanism of the electron transfer from CPR to monooxygenase as follows. Even if the coupling of the redox and the structural states is weak, it could be sufficient for the reduced CPR to initiate the interaction with monooxygenase to which electron is delivered. After the initial weak binding, the binding affinity could become stronger via induced-fit-like structural change of CPR toward the open form as seen in the CPR-HO complex crystal structure. It is noteworthy that the single-protein tracking experiment^26^ detected two binding states, weak and strong binding states, for the association between CPR and cytochrome P450. The principal modes (Fig. 5) suggest that the overall direction toward the structural state suitable for the monooxygenase binding and electron transfer is embedded in the equilibrium thermal fluctuation of CPR.

Then, what physical mechanism can explain the observed coupling of the redox and the structural state? Addressing this question is beyond the scope of the present study, so we just mention what could be the key. The surface of the FMN domain is largely polar, presenting bipolarity^11^. Furthermore, we can find clear electrostatic complementarity between the FMN and the FAD domains, which leads us to expect that the electrostatic attraction between the two domains is the origin of the intrinsic stability of the closed state. Then, the redox state change, which is accompanied by the net charge change, could affect the electrostatic interaction between the FAD and the FMN domains. Binding of NADP^+^, which carries net negative charge, could also affect the interaction between the two domains, triggered by local rearrangement of the electrostatic bonds; Actually, such rearrangement of the electrostatic bonds involving Asp632, which is located near the binding site of NADP^+^ and was noticed in the recent structural studies^27,28^, was observed in our MD simulation (data not shown). In addition, the redox state change in FMN (and also in heme) should affect the electrostatic interaction between the FMN domain and monooxygenase^29^. From the viewpoint of electrostatics, proteins are regarded as dielectric materials. Then, CPR should exhibit dielectric response to the electrostatic inputs (in the present case, the redox-state change and NADP^+^ binding), as was found in the ATP-binding induced dielectric response of myosin^30,31^. The dielectric response causes the polarization charge on the domain surface, affecting the electrostatic interactions between the two domains and between CPR and monooxygenase. The atomic-level analysis for the dielectric response, which is caused by large-scale concerted rearrangement of the electrostatic bonds (called “dielectric allostery^30,31^”), will be done in our next study.

Considering that clear electrostatic complementarity also exists at the interface between CPR and HO^9^ and between CPR and cytochrome P450^19,32,33^, the rearrangement of the electrostatic interaction network in CPR should play the key role in the regulation of downstream protein binding and intermolecular electron transfer. Then, we have to remember that CPR and the downstream proteins are located on the membrane, and the membrane environment exerts substantial influence on the function of these proteins^19,26,32,34^. Collectively taking into account (i) the above-mentioned electrostatic complementarity, (ii) the fact that the membrane surface is largely polar and lipid molecules often contain charged head groups^19^, and (iii) anomalous dielectric property of water that could arise near the surface^35^, it is obviously important to investigate the electrostatic effect of the lipid membrane on the redox-structural state coupling of CPR and the association between CPR and the downstream proteins. MD studies of these proteins in the presence of the membrane^18,19,32,33^ are offering the important first steps toward elucidating the electrostatic effect of the membrane on this electron transfer machinery.

## Methods

As the initial structure of the MD simulation, we used the two crystal structures of CPR, one in the closed form^7^ (PDB ID: 1AMO, chain A), and the other in the open form^8^ (PDB ID: 3ES9, chain A). Missing (or mutationally deleted) residues in the hinge and loop regions were complemented by MODELLER^36^, while the N-terminal transmembrane region (63 residues) were kept removed. The N- and C-termini were capped by the acetyl and the N-methyl-amide groups, respectively. His180, His403, and His615 were doubly protonated according to the evaluation by PROPKA^37^ and H++^38^. The AMBER FF03 force field was used^39^. In this study, we investigated two redox states of CPR: one is the fully oxidized state where FAD and FMN cofactors are both oxidized, and the other is the fully reduced state where FAD and FMN cofactors are both two-electron reduced in the anionic form (FADH^−^ and FMNH^−^)^6^. We obtained the atomic charge parameters for FAD and FMN by AM1-BCC^40^ using antechamber^41^ in combination with the GAFF force field^42^. We used the existing parameter for NADP^+^^43^.

CPR was immersed in a truncated octahedral unit cell filled with 32476 water molecules (TIP3P water^44^) and 150 mM NaCl (113 Na^+^ and 87 Cl^−^ for the oxidized state, and 115 Na^+^ and 87 Cl^−^ for the reduced state), to which the periodic boundary condition was applied. The particle mesh Ewald method^45^ was employed for the calculation of the electrostatic interaction with the real-space cutoff of 8 *Å*^2^. The system was energy-minimized (1000-step steepest descent and subsequent 1000-step conjugate gradient minimizations) with the positional restraints on the main-chain atoms, cofactors, and the side-chain atoms of the residues within 10 *Å*^2^ from the cofactors (the restoring force constant was set at 500 kcal/mol/*Å*^2^), and further energy-minimization (2000 steps) was carried out without restraints. The system was then heated to 310 K under the constant volume condition for 50 ps, during which the atoms that were restrained in the minimization were again restrained, with the restoring force gradually reduced from 10 to 0.01 kcal/mol/*Å*^2^. The volume of the system was then allowed to relax at 1.0 × 10^5^ Pa for 1 ns. For the temperature control, the Langevin dynamics was used^46^ with the collision frequency of 1 ps^−1^. For the pressure control, the Berendsen barostat^47^ was used with the relaxation time constant of 50 ps (5 ps for the subsequent production runs). The time step was set to 2 fs by applying the SHAKE method^48^.

We then conducted production runs: 16 independent 0.2-μs runs for each redox state (oxidized or reduced) starting from the closed form, and 8 independent 0.2-μs runs for each redox state starting from the open form. To accelerate the molecular dynamics, we further conducted 1-μs accelerated MD (aMD)^20^ subsequent to each conventional MD (cMD) run starting from the closed form and 0.4-μs aMD subsequent to each cMD run starting from the open form (therefore the total simulation lengths for cMD and aMD are 4.8 μs and 19.2 μs, respectively, for each redox state). In the aMD, we used the boosted potential energy V* for the dihedral and the total potential energies^20^, i.e.,

1 $$\begin{array}{rcl}{V}^{\ast }({\boldsymbol{r}}) & = & V({\boldsymbol{r}})+{\rm{\Delta }}V({\boldsymbol{r}}),\\ {\rm{\Delta }}V({\boldsymbol{r}}) & = & \{\begin{array}{ll}0 & (V({\boldsymbol{r}})\ge {E}_{{\rm{B}}})\\ \frac{{({E}_{{\rm{B}}}-V({\boldsymbol{r}}))}^{2}}{\alpha +{E}_{{\rm{B}}}-V({\boldsymbol{r}})} & (V({\boldsymbol{r}}) < {E}_{{\rm{B}}})\end{array}\end{array}$$

where *V*, *E*_B_, and *α* denote the original potential energy, the threshold energy below which boosting (energy lifting) is turned on, and the parameter to determine the extent of boosting, respectively. According to the prescription^49^, we set ${E}_{{\rm{B}}}=0.091 \sim 0.093\times {10}^{5}$ kcal/mol and $\alpha =0.005\times {10}^{5}$ kcal/mol for the dihedral potential boost, and ${E}_{{\rm{B}}}=-\,3.222 \sim -\,3.239\times {10}^{5}$ kcal/mol and $\alpha =0.172\times {10}^{5}$ kcal/mol for the total potential boost. To obtain the canonical ensemble from the aMD trajectory, the statistical weight of an aMD snapshot is multiplied by $\exp ({\rm{\Delta }}V/{k}_{{\rm{B}}}T)$ using the Δ*V* value for the snapshot^20^ (*k*_*B*_ is the Boltzmann constant and *T* = 310 K). The volume of the system was kept constant in the aMD period in consideration of the reduced virial due to the boost potential. To investigate the effect of the bound NADP^+^, we also conducted MD simulations in the absence of NADP^+^ (16 runs of 0.2-μs cMD followed by 1-μs aMD) for each redox state. All of the MD simulations were executed using AMBER12^50^. All of the MD simulations that we conducted in the present study were summarized in Table 1.

**Table 1:** MD runs conducted in this study.

Redox state	Initial structural state	MD method	MD length (μs)	#MD runs	Total length (μs)
Oxidized	Closed	cMD	0.2	16	19.2
		aMD	1.0	16	
Reduced	Closed	cMD	0.2	16	19.2
		aMD	1.0	16	
Oxidized	Open	cMD	0.2	8	4.8
		aMD	0.4	8	
Reduced	Open	cMD	0.2	8	4.8
		aMD	0.4	8	
Oxidized (no NADP^+^)	Closed	cMD	0.2	16	19.2
		aMD	1.0	16	
Reduced (no NADP^+^)	Closed	cMD	0.2	16	19.2
		aMD	1.0	16	
